# Downregulation of Zn-transporters along with Fe and redox imbalance causes growth and photosynthetic disturbance in Zn-deficient tomato

**DOI:** 10.1038/s41598-021-85649-w

**Published:** 2021-03-16

**Authors:** Ahmad Humayan Kabir, Mst Salma Akther, Milan Skalicky, Urmi Das, Gholamreza Gohari, Marian Brestic, Md. Monzur Hossain

**Affiliations:** 1 Department of Botany, University of Rajshahi, Rajshahi, 6205 Bangladesh; 2 Department of Botany and Plant Physiology, Faculty of Agrobiology, Food and Natural Resources, Czech University of Life Sciences Prague, Kamycka 129, 165 00 Prague, Czech Republic; 3 Department of Horticultural Sciences, Faculty of Agriculture, University of Maragheh, Maragheh, Iran; 4 Department of Plant Physiology, Slovak University of Agriculture, Nitra, Tr. A. Hlinku 2, 94901 Nitra, Slovakia

**Keywords:** Plant sciences, Photosynthesis, Plant physiology

## Abstract

Zinc (Zn) deficiency hinders growth and development in tomato. This study unveils the responses of how Zn starvation affects physiological and molecular processes in tomato. Zn deficiency negatively affected the biomass, cellular integrity, and chlorophyll synthesis in tomato. Also, Zn deficiency decreased the maximum yield of PSII, photosynthesis performance index and dissipation energy per active reaction center, although the antenna size, trapping energy efficiency and electron transport flux were stable in Zn-starved leaves. Further, Zn shortage caused a substantial reduction in Zn and Fe concentrations in both roots and shoots along with decreased root Fe-reductase activity accompanied by the downregulation of *Fe-regulated transporter 1*, *Zn transporter-like (LOC100037509)*, and *Zn transporter (LOC101255999)* genes predicted to be localized in the root plasma membrane. The interactome partners of these Zn transporters are predominantly associated with root-specific metal transporter, ferric-chelate reductase, *BHLH* transcriptional regulator, and Zn metal ion transporters, suggesting that Zn homeostasis may be tightly linked to the Fe status along with *BHLH* transcription factor in Zn-deficient tomato. We also noticed elevated O_2_^.−^ and H_2_O_2_ due to Zn deficiency which was consistent with the inefficient antioxidant properties. These findings will be useful in the downstream approach to improve vegetable crops sensitive to Zn-deficiency.

## Introduction

Zinc (Zn) shortage, a scarcity of plant nutrition has an inauspicious consequence on vegetables^1^. With high pH or alkaline stress, there are many lands in different regions across the globe are Zn-deficient^2^. The soil, which contains a sandy texture, as well as less phosphorus, is also deficient in Zn^3^. The plants which are sensitive Zn-starved conditions are often exhibiting reduced growth, dwarf stems, and light chlorosis^4^. The functions of Zn can be found in photosynthetic and gene expression processes besides enzymatic and catalytic activities^5^. Inadequacy of Zn also resulted in a deterioration in plant stomatal activity^1^. In response to the pathogenic invasion of plants, Zn proteins also play a significant role^6^. The insufficiency of Zn in plant-based foods contributes to human malnutrition throughout the world^7^.

While generally there is a genotypic difference in Zn-deficiency responses, a plethora of plants are susceptible to Zn deprivation^4,8^. As Zn efficiency mechanisms are somewhat complex in their actions, research has been found a few adaptive features confer Zn deficiency in plants^4,9^. Since it is tough for Zn to penetrate the root cell membranes, therefore the elevation of Zn is attained through transporters at the primary stage^4,8^. The *IRT1*, known as Fe-regulated transporter, is often incited to ameliorate translocation as well as Zn exploitation whenever the plant has possibly Zn or Fe shortcomings^10,11^. *IRT1* is responsible for transporting several minerals such as Zn, Mn, and Fe, even though the pattern of expression varies depending on mineral stresses^12^. Fe and Zn are strictly regulated in plants and they are typically connected owing to the chemical resemblance and *IRT1* non-specificity^13^. Besides, ferric chelate reductase (FCR) activity along with Strategy I responses are induced in Fe-deprived plants, which are generally dicots^14,15^. FCR affects the availability of Fe and could influence the absorption of different types of metals^16^. Hereafter, improvement is declared to conferring the involvement of *ZIP* (Zn transporter) in Fe and Zn uptake^11,17,18^, though the evidence in tomato continues to be limited. Pavithra et al.^19^ classified as low-affinity (*SlZIP5L2*, *SlZIP5L1*, *SlZIP4*, and *SlZIP2*) and high-affinity (*SlZIPL*) Zn transporters in tomato. Nevertheless, these explications were tentatively claimed in the shoot instead of root tissue, in which the absorption of metal occurs in plants.

The deficiency of Zn also causes the accumulation of excessive reactive oxygen (ROS) species, which impedes photosynthesis and protein biosynthesis^4,9^. Therefore, elevated detoxifying ROS is one of the pioneer strategies plants often possess in response to abiotic stress. ROS scavenging enzymes play a vital part in controlling reactive radicals to restore the cell's redox balance^9^. Among the enzymes, catalase (CAT) and superoxide dismutase (SOD) were mainly demonstrated to underlie ROS scavenging in Zn-deprived plants^20^. Besides, ascorbate peroxidase (APX) is able to transform excessive H_2_O_2_ into the water in ROS-induced plants^21^. However, the induction of antioxidant resistance largely depends on the plant species/genotypes.

Tomato (*Solanum lycopersicum* L.) is known to a high-value crop all over the world^22^. The insufficiency of the inevitable micronutrient Zn adversely affects tomato production worldwide. However, the outcome of low Zn availability on the physiological and molecular repercussions for which tomato plants suffer from growth retardation remains entirely unfamiliar. In this manner, this study aims at unveiling the reasons for dysfunctions found in the tomato plant under Zn deficiency supported by the different biochemical and molecular investigations.

## Materials and methods

### Plant cultivation technique

As obvious from a previous study^23^, Zn-sensitive tomato cultivar (cv. Ratan) was used for this study. The periphery of the seeds was cleaned up with 70% ethyl alcohol for 3 min, followed by doubled Milli-Q water before transferred to the germination tray at room temperature. The sprouted three-days homogeneous plantlets thereafter were transferred to the hydroponic solution at pH 6.0^24^, as it is formerly narrated (µM): KNO_3_ (1600), Ca(NO_3_)_2_.4H_2_O (600), KH_2_PO_4_ (100), MgSO_4_.7H_2_O (200), KCl (50), H_3_BO_3_ (25), Fe-EDTA (20), MnSO_4_.4H_2_O (2), Na_2_MoO_4_.2H_2_O (0.5) and CuSO_4_.5H_2_O (0.5). Furthermore, ZnSO_4_ has been used as follows^8^: 2.0 µM (Zn-sufficient or control) and 0.01 µM (Zn-deficient). The pH of the solution was adjusted to 6.0 using KOH/HCl and the solution was changed in every 4 days interval. The seedlings were grown in an aerated plastic container (1.2 L) for each treatment having 9 plants/container in an indoor growth chamber. Each seedling was inserted through foam attached to a cut centrifuge tube, exposing the roots to the solution. The growth chamber was strictly controlled with photoperiod 14 h dark/10 h light at ± 26 °C, 65% air humidity, and 200 μmol m^−2^ s^−1^ light intensity. The plants were cultivated for 14d before harvesting for data analysis.

### Characterization of morphology, photosynthesis and Fe chelate reductase activity

A digital caliper was used for measuring the length of the root and shoot. The dry weight of root and shoot was recorded after drying for 3 days at 80 °C in an electric oven. For measuring the chlorophyll score of young leaves, a SPAD meter was used (Minolta, Japan). Furthermore, photosynthesis biophysics through chlorophyll fluorescence kinetic (OJIP), such as maximum quantum yield of PSII (FvFm), photosynthesis performance index (Pi_ABS), dissipation energy per active reaction center (DIo/RC), absorption flux/effective antenna size of an active reaction center (ABS/RC), electron transporter flux further than QA DIo/RC (ET2o/RC) and trapped energy flux leading to a reduction of QA (TRo/RC) was recorded on young leaves kept for 1 h at dark using FluorPen FP 100 (Photon Systems Instruments, Czech Republic).

Fe chelate reductase activity (FCR) activity in roots was determined in roots by ferrozine assay^15^. Briefly, the 0.2 mM CaSO_4_ followed by Milli-Q water washed off the root surface. The roots samples were then homogenized with 1 ml of assay mixture (100 mM Fe(III) EDTA, 0.10 mmol MES-NaOH (pH 5.5), 300 mM ferrozine). The samples and blank tubes (without assay mixture) were incubated in the dark for 20 min at 25 °C. Finally, aliquots were read at 562 nm. The FCR activity was determined by using the molar extinction coefficient (27.9 mM^−1^ cm^−1^) of ferrozine.

### Elemental analysis in root and shoot

Tomato roots were rinsed out once with 0.1 mM CaSO_4_ and several instances with Milli-Q water to eliminate external components. The roots and shoots were then desiccated individually at 70 °C in an oven for 3d. The samples were then digested with HNO_3_/HClO_4_, 3:1 v/v. Afterward, the Zn and Fe were tested by means of atomic absorption spectroscopy (AA-6800, SHIMADZU).

### Determination of soluble protein

In brief, fresh root and shoot were ground with tris–HCl buffer (50 mM, pH 7.5), 2 mM EDTA and 0.04% (v/v) β-mercaptoethanol. In order to separate the transparent fluid portion, the crude specimens were centrifuged for 10 min at 12,000 rpm (MicroCL 21, Thermo Scientific, United States). Subsequently, 1 ml of Coomassie Brilliant Blue (CBB) was diluted to 100 µl protein extract before measuring the absorbance at 595 nm. A bovine serum albumin (BSA) curve was modeled for estimating the total soluble protein as described by Bradford assay^25^.

### Assessment of electrolyte leakage and cell death

The ultimate consequence of damage of the cell membrane integrity in both root and shoot were measured by using a conductivity meter with some modifications^26^. Root and shoot surface components were washed with deionized water. Thereafter, the freshly harvested samples were transferred into a beaker filled with 20 ml deionized water and kept at 25 °C for 2 h. Later, the solution’s electrical conductivity (EC1) was calculated. Afterward, the samples were heated in a water bath for 20 min at 95 °C then soothed at 25 °C before recording the final electrical conductivity (EC2). The electrolyte leakage was then determined as follows: = (EC1/EC2) × 100 (%).

Evans blue was determined to account for the cell death rate with some modifications^27^. The entire fresh root and shoot were placed into 2 mL of Evan's blue mixture for 15 min. The samples were then mixed with 80% ethyl alcohol and placed at room temperature for 10 min. Afterward, the solutions were incubated in a water bath for 15 min at 50 °C and were further centrifuged for 10 min at 12,000 rpm (MicroCL 21, Thermo Scientific, United States). The supernatant was then transferred into a new centrifuge tube before measuring the absorbance at 600 nm. Eventually, the % of cell death was calculated.

### Analysis of O_2_^−^, H_2_O_2_ and lipid peroxidase

The superoxide (O_2_^−^) was tested as mentioned previously^28^. Briefly, the fresh samples (0.1 g) were washed with water, homogenized with chilled K-phosphate buffer (10 mM), and centrifuged at 12,000 rpm at 4 °C for 10 min. The clear supernatant (100 µl) was mixed up with 1 ml of assay solution with 0.5 mM XTT sodium salt and 50 mM Tris–HCl (pH 7.5). Finally, the solution's absorbance was read at 580 nm and the superoxide (O_2_^−^) was calculated by the coefficient of extinction 2.16 × 104 M^−1^ cm^−1^). The H_2_O_2_ accumulation was measured, utilizing 0.1% C_2_HCl_3_O_2_^29^. The fresh samples (0.1 g) were ground with a mortar and pestle in 0.1% C_2_HCl_3_O_2_ and further centrifuged at 10,000 rpm for 15 min. The top aqueous segment (100 µl) was appended with assay solution supplemented with 10 mM K-phosphate (pH 7.0), 1 M KI. The samples were then adapted to dark for 1 h before recording the absorbance at 390 nm (Shimadzu UV-1650PC). For the determination of lipid peroxidase activity, the fresh samples (0.1 g) were homogenized in 20% C_2_HCl_3_O_2_ incorporated with 0.5% C_4_H_4_N_2_O_2_S mixture. The clear supernatant (1 ml) was warmed at 95 °C for 30 min in a water bath and subsequently chilled on ice. The samples were then centrifuged at 12,000 rpm for 10 min. The optical density of the samples was monitored 532 and 600 nm (60S UV–Visible Spectrophotometer, Thermo Scientific, United States). The lipid peroxidase activity was determined according to the coefficient of molar extinction 155 mmol L^−1^ cm^30^.

### Gene expression and bioinformatics analysis

The total RNA was isolated from the fresh roots as described by SV total RNA isolation system (Promega, USA). The quantified RNA was then converted to cDNA using the cDNA synthesis kit (Promega, USA) before performing real-time PCR analysis in an ECO real-time PCR system (Illumina, USA) using gene-specific primers for iron-regulated transporter 1, zinc transporter-like (*LOC100037509*) and zinc transporter (*LOC101255999*) (Table S1). The PCR reactions were set as follows: 95 °C for 3 min, followed by 40 cycles at 95 °C for 10 s, 57 °C for 30 s. The relative expression of candidate genes was calculated by the dd − ∆Ct method considered *Actin* as an internal control. Further, the sub-cellular localization of these genes was predicted by CELLO v.2.5. The interactome networks of iron-regulated transporter 1, zinc transporter-like (*LOC100037509*) and zinc transporter (*LOC101255999*) were generated based on the physical interactions among molecules (protein–protein interactions) using the STRING server (http://string-db.org) visualized in Cytoscape^31^.

### Activities of antioxidant enzymes

In brief, the root and shoot samples (0.1 g) were independently homogenized with 100 mM phosphate (pH 7.0) in a mortar and pestle. The homogenate was centrifuged at 8000 rpm for 10 min (MicroCL 21, Thermo Scientific, United States)., and the transparent portion was collected for analysis. We first analyzed the SOD activity by mixing the plant extract (100 µl) with 0.1 mM of EDTA, 50 mM of NaHCO_3_ (pH 9.8), and 0.6 mM of epinephrine^32^. After 4 min, the adrenochrome confirmation was read at 475 in a spectrophotometer. Secondly, the plant extract (100 µl) was supplemented with 1 ml of assay solution with 0.1 mM EDTA, 0.1 mM H_2_O_2_, 0.5 mM ascorbic acid, and 50 mM phosphate^33^. The absorbance of the mixture was then observed at 290 nm and thereafter calculated for APX activity by extinction coefficient (2.8 mM^−1^ cm^−1^). Besides, 100 µl extract was mixed up with 100 mM phosphate buffer and 6% hydrogen peroxide solution. The absorbance was read twice in 30 s interval and thereafter calculated for the activity of CAT using a coefficient of 0.036 mM^−1^ cm^−1^). Lastly, 100 mM phosphate, 1 mM EDTA, 20 mM GSSG and 0.2 mM NADPH added individually to 100 μL of plant extract. The reaction was initiated by GSSG, and absorption was reduced by NADPH-oxidation at 340 nm. The calculation of GR activity was then performed by the extinction coefficient (6.12 mM^−1^ cm^−1^)^34^.

### Statistical analysis

We opted for a completely randomized block design (CRBD) in plant cultivation. Three independent biological replications were considered for data analysis. Each experiment was performed at least three times to check whether the results were reproducible. The statistical significance of mean was evaluated by Microsoft Excel 2010 at *p* ≤ 0.05 by *t*-test. Figures were plotted by using GraphPad Prism (V. 6.0) software.

## Results

### Changes in morphology, photosynthetic parameters and FCR activity

**Figure 1:**
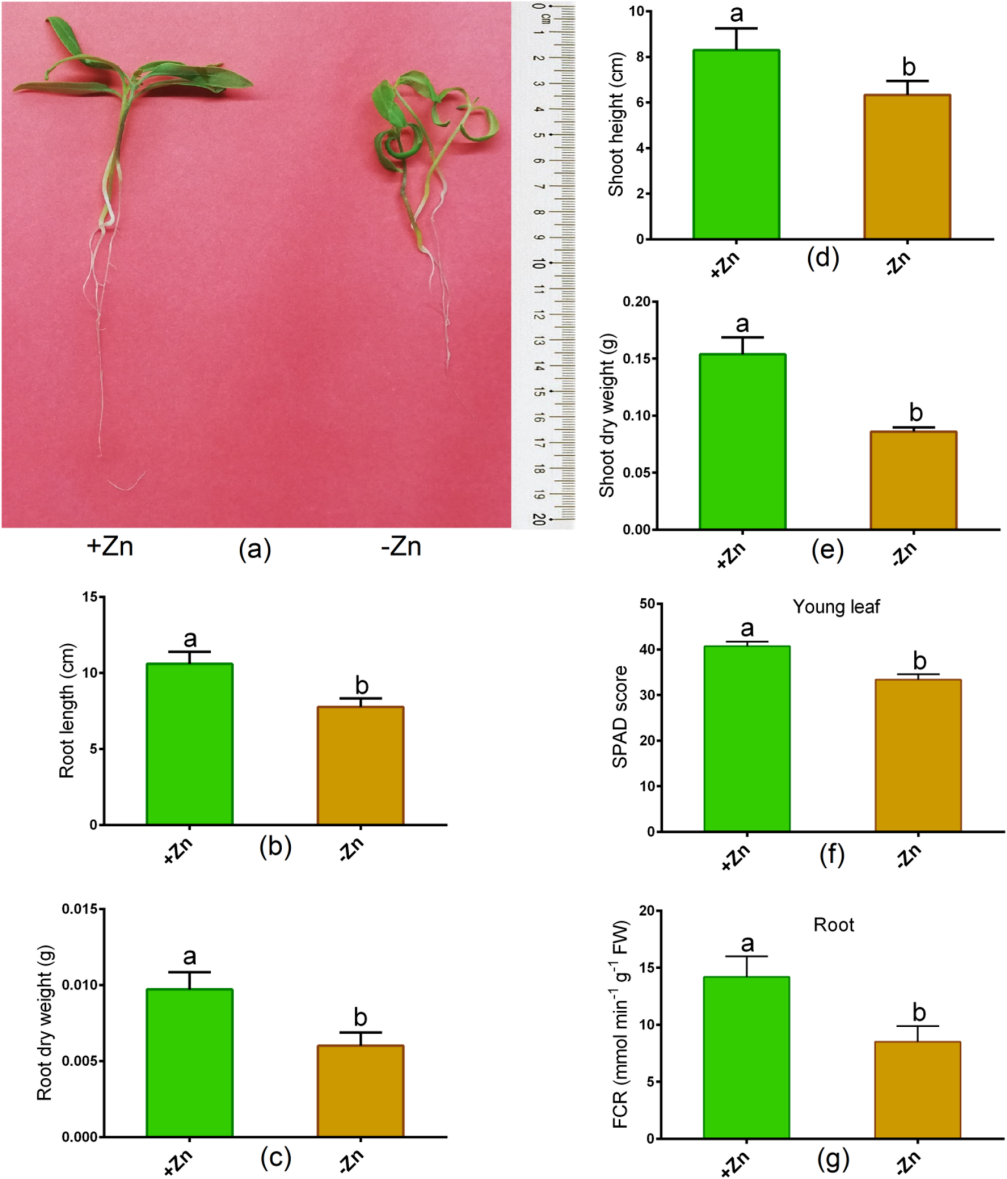
Phenotype (**a**), root length (**b**), root dry weight (**c**), shoot height (**d**), shoot dry weight (**e**), leaf chlorophyll score (**f**) and root FCR (ferric chelate reductase) activity (**g**) in tomato cultivated under Zn-sufficient and Zn-deficient conditions for 14 days. Different letters indicate significant differences between means ± SD of treatments (*p* < 0.05, n = 3).

**Figure 2:**
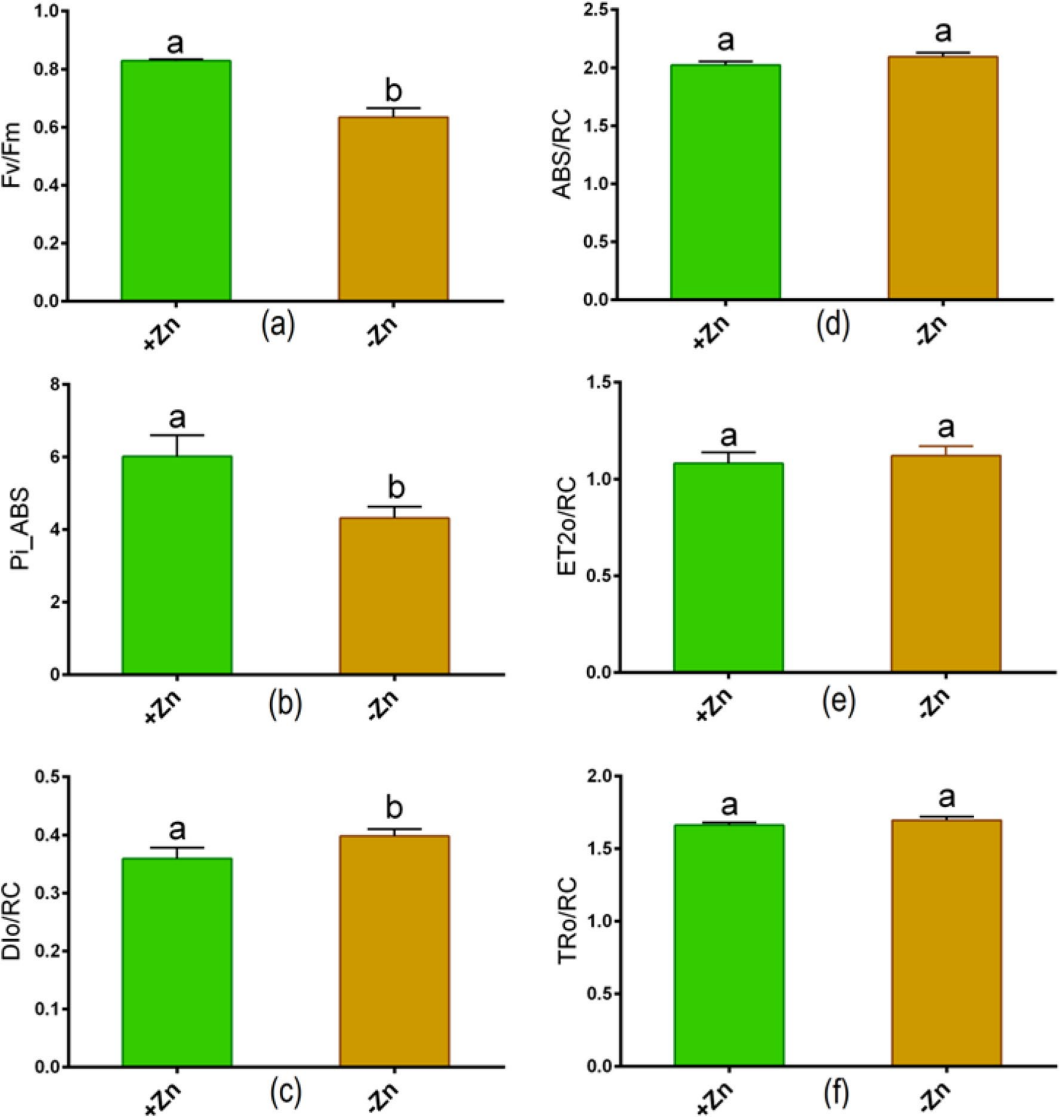
Chlorophyll fluorescence parameters: (**a**) maximum quantum yield of PSII (Fv/Fm), (**b**) photosynthesis performance index (Pi_ABS), (**c**) dissipation energy per active reaction center (DIo/RC), (**d**) absorption flux/effective antenna size of an active reaction center (ABS/RC), (**e**) electron transport flux further than QA DIo/RC (ET2o/RC) and (**f**) trapped energy flux leading to a reduction of QA (TRo/RC) in young leaves of tomato cultivated Zn-sufficient and Zn-deficient conditions for 14 days. Different letters indicate significant differences between means ± SD of treatments (*p* < 0.05, n = 3).

**Figure 3:**
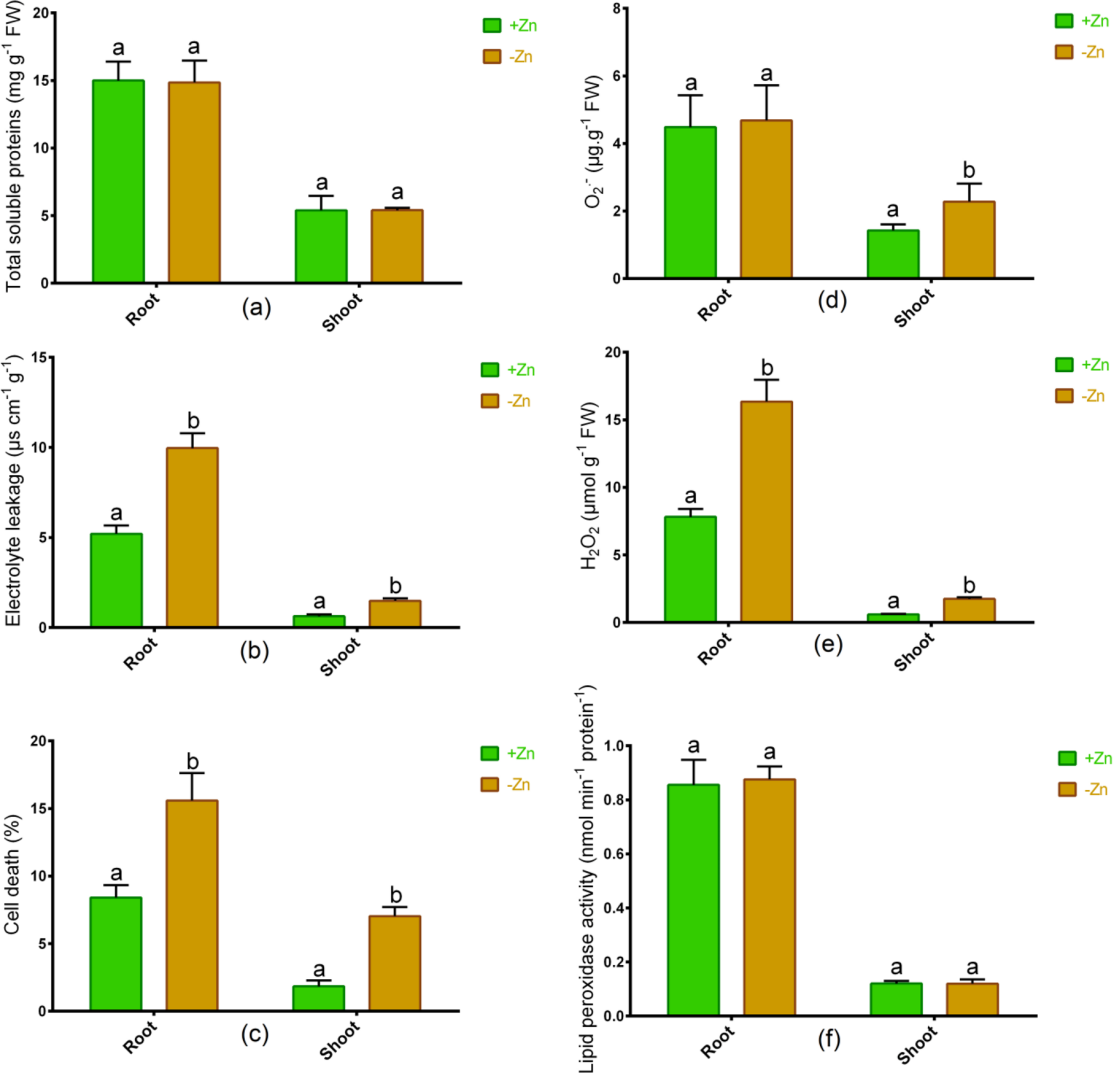
Total soluble proteins (**a**), electrolyte leakage (**b**), cell death % (**c**), O_2_^−^ (**d**), H_2_O_2_ (**e**) and lipid peroxidase activity (**f**) in roots and shoots of tomato cultivated under Zn-sufficient and Zn-deficient conditions for 14 days. Different letters indicate significant differences between means ± SD of treatments (*p* < 0.05, n = 3).

Zn deficiency showed a significant reduction in the root (root length, dry root weight) and in the shoot characteristics (shoot height, dry shoot weight) in Ratan than untreated controls (Fig. 1a–e). In addition, Zn-deprivation led to a significant reduction in the SPAD score as opposed to control plants (Fig. 3a). Besides, the leaf chlorophyll score and root FCR activity significantly decreased owing to Zn-deprivation relative to controls (Fig. 1f,g). The Fv/Fm and Pi_ABS values showed a large decrease in young leaves as a result of Zn-shortage as opposed to controls (Fig. 2a,b). However, the DIo/RC value considerably increased in Zn-starved leaves in contrast to controls (Fig. 2c). OJIP analysis showed no changes in ABS/RC, ET2o/RC, and TRo/RC values between Zn + and Zn− conditions (Fig. 2d–f).

### Changes in stress indicators

In Ratan, the total soluble protein in the root and shoot remained unchanged due to Zn-deprivation in contrast to plants grown with optimal Zn (Fig. 3a). Furthermore, cell death (%) and electrolyte leakage in either root or shoot increased significantly following Zn shortage relative to controls in Ratan (Fig. 3b,c). Although the O_2_^−^ showed no changes in the root, the accumulation of this ROS significantly increased following Zn starvation relative to Zn-sufficient plants (Fig. 3d). The H_2_O_2_ in root and shoot of tomato significantly increased owing to Zn-deficiency relative to controls (Fig. 3e). However, the lipid peroxidase activity remained unchanged between Zn-sufficient and Zn-deficiency conditions (Fig. 3f).

### Metal analysis, gene expression pattern and in silico validation

**Figure 4:**
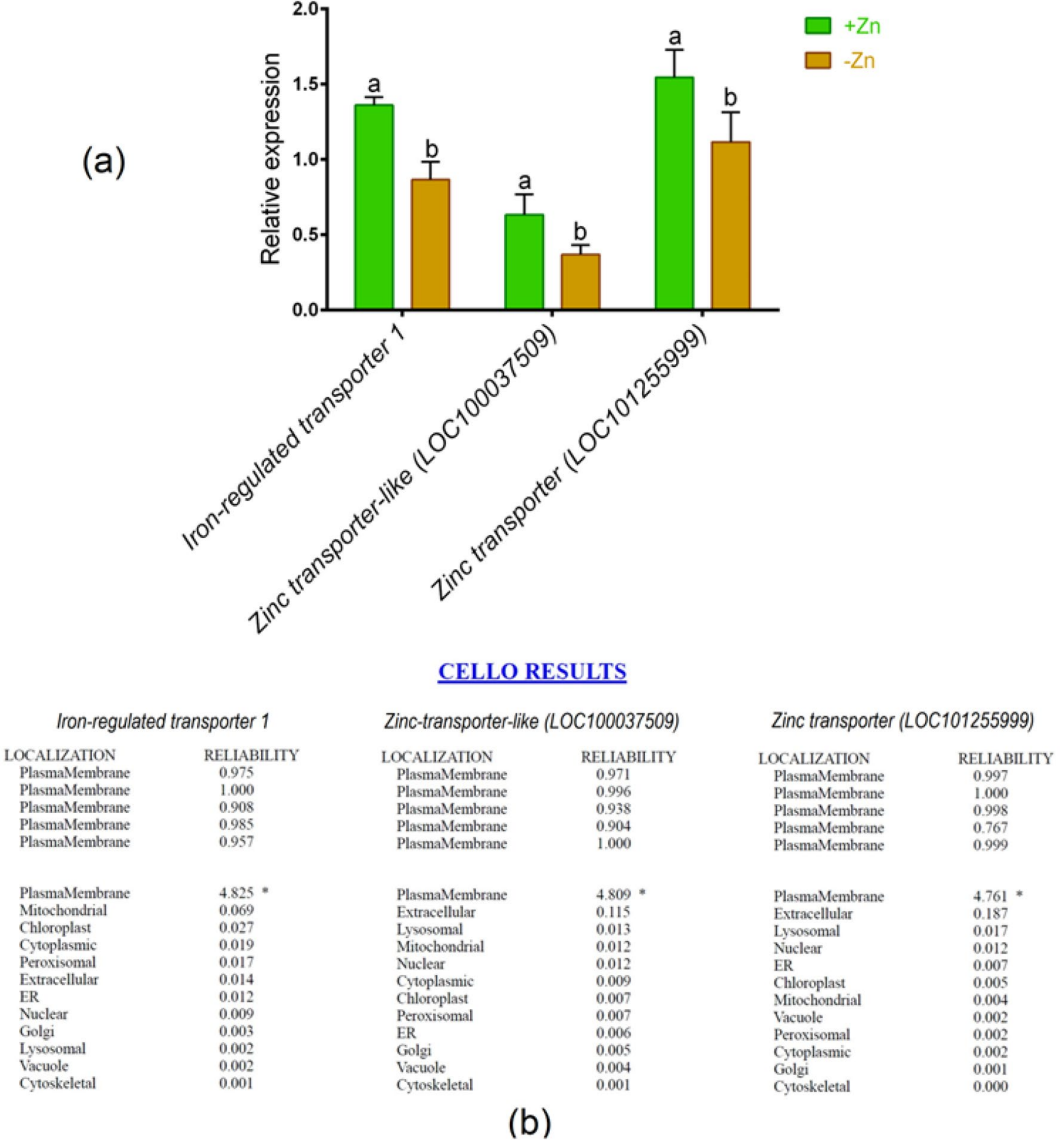
Quantitative expression of iron-regulated transporter 1*,* zinc transporter-like *(LOC100037509*) and zinc transporter (*LOC101255999*) genes in roots of tomato cultivated under Zn-sufficient and Zn-deficient conditions for 14 days (**a**) and computational sub-cellular localization prediction (**b**). Different letters indicate significant differences between means ± SD of treatments (*p* < 0.05, n = 3).

The concentrations of Fe and Zn showed a significant decrease in root and shoot because of Zn insufficiency relative to Zn-sufficient plants (Table 1). To validate the metal analysis, we analyzed the expression pattern of key Zn transporters in tomato roots. In real-time PCR analysis, Fe-regulated transporter 1 showed a substantial downregulation in roots subjected to Zn-shortage as opposed to Zn-sufficient plants (Fig. 4a). Additionally, the expressions of Zn transporter-like (*LOC100037509*) and Zn transporter (*LOC101255999*) genes were significantly downregulated due to Zn shortage in roots compared with Zn sufficient controls (Fig. 4a).

**Table 1:** Zn and Fe concentrations (mg kg^−1^) in roots and shoots of tomato cultivated under Zn-sufficient and Zn-deficient hydroponic conditions for 14 days.

Parameters	Root		Shoot	
	+ Zn	−Zn	+ Zn	−Zn
Zn	217.7 ± 17.8a	135.17 ± 14.1b	127.2 ± 17.1a	69.9 ± 12.8b
Fe	254.1 ± 21.3a	117 ± 16.1b	101.6 ± 12.2a	38.6 ± 9.7b

Different letters indicate significant differences between means ± SD of treatments (*p* < 0.05, n = 3).

**Figure 5:**
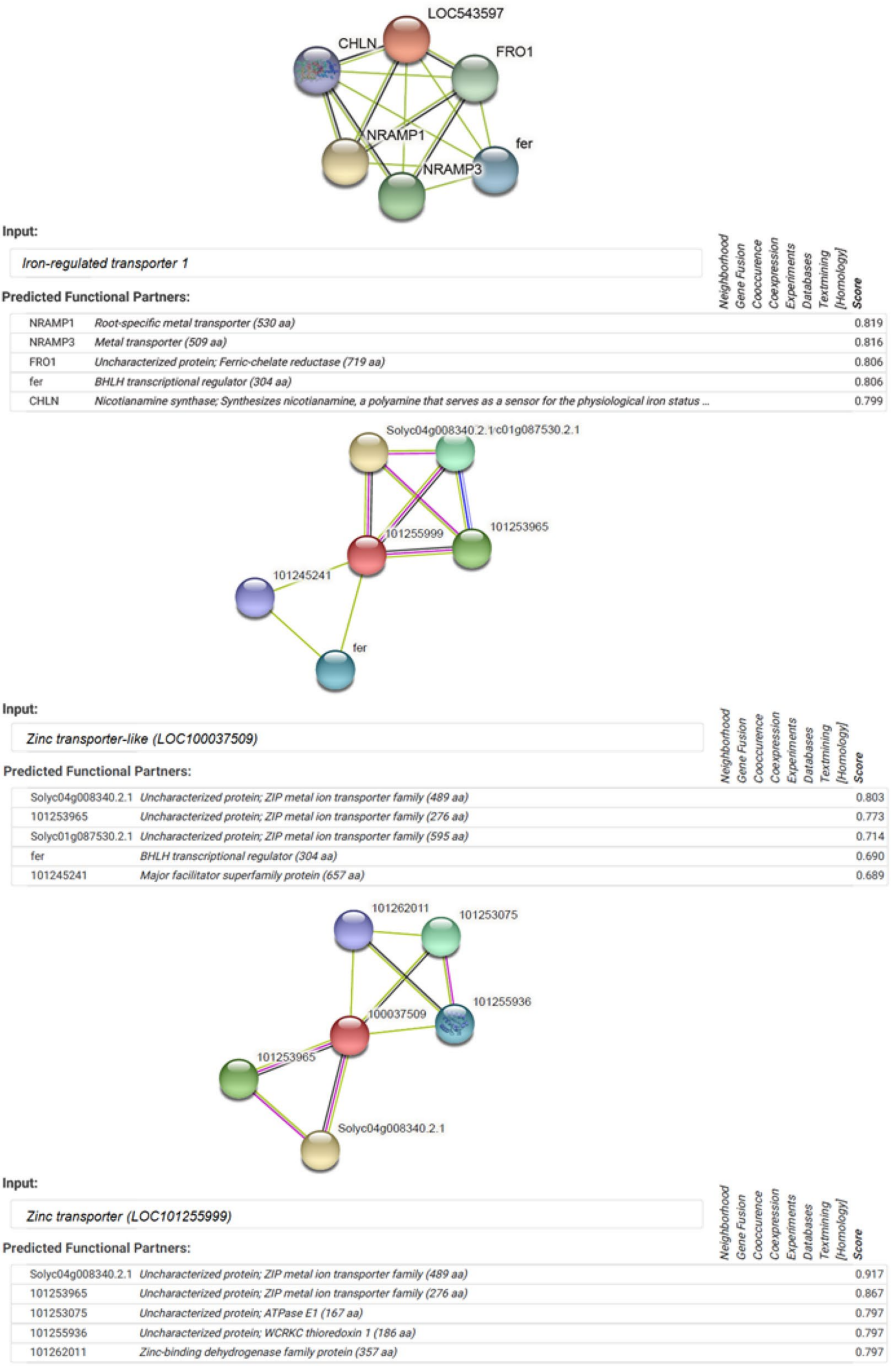
Predicted gene interaction partners of iron-regulated transporter 1, zinc transporter-like *(LOC100037509*) and zinc transporter (*LOC101255999*) genes in tomato. Interactome was generated using Cytoscape for STRING data.

CELLO predictor showed that these genes are localized in the plasma membrane of the root resulting highest reliability score (Fig. 4b). STRING showed five putative interaction partners of iron-regulated transporter 1, which include *NRAMP1* (root-specific metal transporter), *NRAMP3* (metal transporter), *FRO1* (ferric-chelate reductase), *fer* (BHLH transcriptional regulator) and *CHLN* (nicotianamine synthase) genes (Fig. 5). In addition, zinc transporter-like (LOC100037509) are closely linked in a partnership with *Solyc05g008340.2.1* (ZIP metal ion transporter family), *101,253,965* (ZIP metal ion transporter family), *101,253,075* (ATPase EI), *101,255,936* (WCRKC thioredoxin) and *101,262,011* (Zn-binding dehydrogenase family protein). Interactome analysis also showed close interactions of Zn transporter (LOC101255999) with *Solyc05g008340.2.1* (ZIP metal ion transporter family), *101,253,965* (ZIP metal ion transporter family), *Solyc01g087530.2.1* (ZIP metal ion transporter family), *fer* (BHLH transcriptional regulator) and *101,245,241* (major facilitator superfamily protein) genes (Fig. 5).

### Changes in antioxidant enzymes

**Figure 6:**
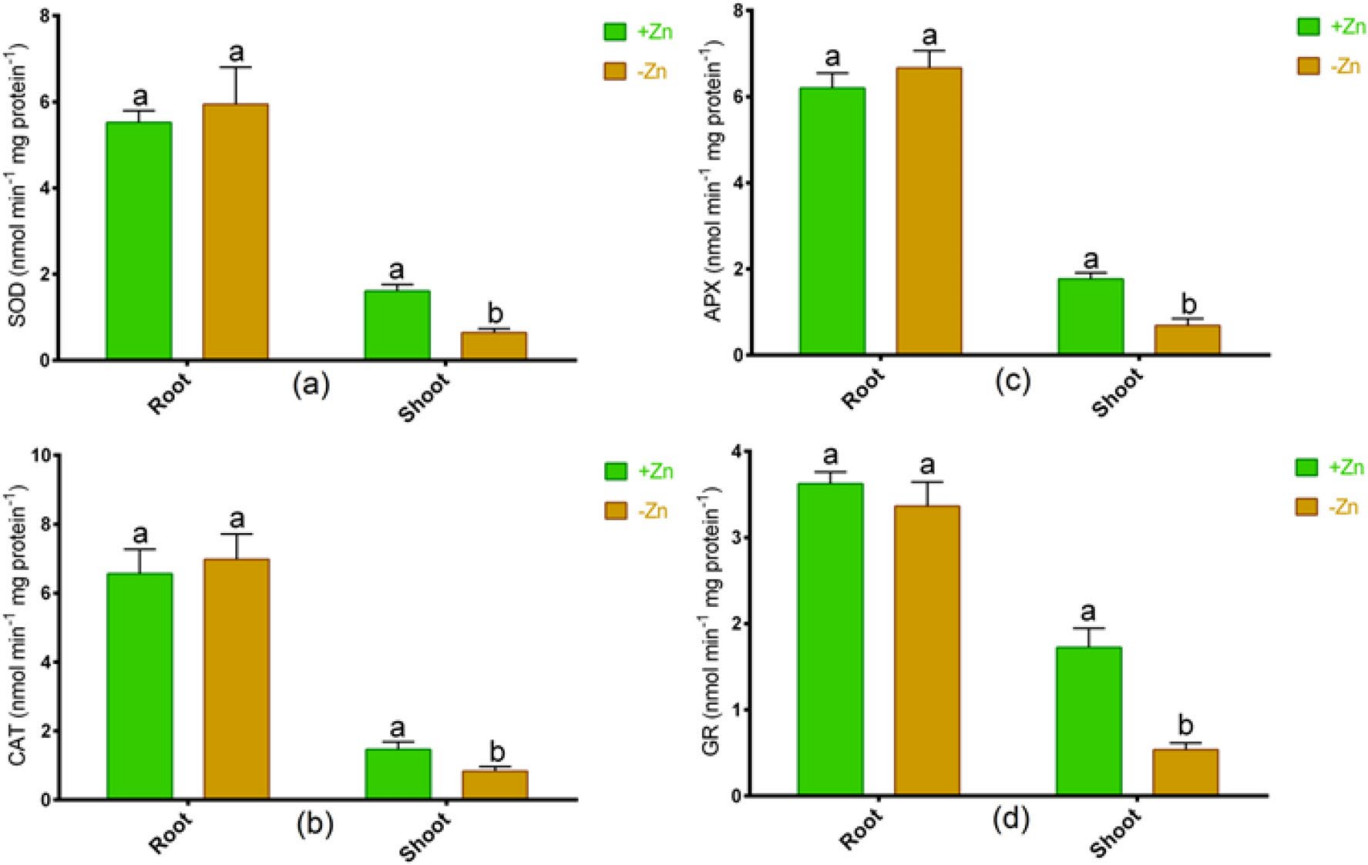
The activities of (**a**) superoxide dismutase (SOD), (**b**) catalase (CAT), (**c**) ascorbate peroxidase (APX) and (**d**) glutathione reductase (GR) in roots and shoots of tomato cultivated under Zn-sufficient and Zn-deficient conditions for 14 days. Different letters indicate significant differences between means ± SD of treatments (*p* < 0.05, n = 3).

We have analyzed the different antioxidant enzymes, whether this tomato cultivar does have antioxidant properties to withstand oxidative damage. Results showed that CAT, SOD, APX, and GR activities remained unchanged in roots, but these enzymes significantly reduced due to Zn-starvation in comparison with Zn-sufficient plants (Fig. 6a–d).

## Discussion

Understanding the mechanistic consequence of mineral deficiency responses is of great importance to initiate crop improvement steps through breeding or genome editing approaches. Long ago^35^, gave a tentative sign that tomato Zn uptake is restricted to the root system rather than the aerial part. Since then, extensive studies related to Zn-starved tomato have not been elucidated. This research offers some new insight into improvements in photosynthesis, Zn transporters, and the antioxidant ability for which tomato plants cannot overcome Zn deficiency.

### Morphological and physiological responses

Zn is essential for metabolism and plant growth. Nevertheless, in Zn inefficient Ratan, the absence of adaptive systems to fight Zn deficiency decreased the root and shoot morphological characteristics. This result is consistent with earlier accounts of Zn deficiency in crop plants^8^. In the research of ours, reduced chlorophyll, PSII capability, and photosynthesis performance index were correlated with the morphological features in Ratan. The chlorophyll value decreases along with Fv/Fm and Pi ABS, indicating that Ratan plants were unable to regulate PSII mechanisms under Zn deficiency. Furthermore, the increase of DIo/RC suggests that Zn-deficiency results in ineffective energy management, which is correlated with the reduced photosynthetic efficiency in leaves of Zn-deprived tomato. Zn functions during photosynthesis when CO_2_ is fixed, although chloroplast and photosynthesis can be impaired under deficiency^36,37^. Several cellular dysfunctions and damages related to plant abiotic stress. In this particular research, while low Zn did not affect overall soluble protein-rich, Ratan displayed a tremendous rise in cell death as well as membrane damage and accumulation of H_2_O_2_ in reaction to Zn deficiency. Membrane leakage and cell death are typical effects of abiotic stress^8,38^. Therefore, our results suggest that an inadequate supply of Zn is one of the features for which Ratan cannot maintain the cellular integrity, morphological, and photosynthesis efficiency subjected to Zn deficiency.

### Changes of Zn and Fe status

In this study, the concentration of Zn and Fe were simultaneously reduced in response to Zn deficiency in tomato, indicating a complicated Zn and Fe homeostasis response. The rapid decrease of root and shot Zn and Fe concentration in Ratan shows that Zn homeostasis cannot be held under Zn hunger by this genotype. Differential tissue Zn levels have been displayed in a few species of plants that are different in response to Zn scarcity^39^. In the cytosol, *IRT* and *ZIP* proteins are mostly associated with Zn delivery^40,41^. Previous studies demonstrate that Zn deficiency also caused Fe deficiency as plants prevent the transfer of Fe from root to shoot^42,43^. Pavithra et al.^19^ emphasized the involvement of shoot showing differential expression of Zn transporters. However, it is slightly confusing as most of the Zn-acquisition genes are localized in the plasma membrane of roots as predicted by the localization predictor. We have therefore carried out a focused analysis on the pattern of expression of *IRT* and *ZIP* in tomato roots. Fe-regulated carrier, Fe-regulated transporter 1, has shown major downregulation in the roots of Ratan, inducing its susceptibility to Zn deficiency. Originally, *IRT1* was identified as a Fe transporter, but complementation and uptake studies revealed its involvement in Mn and Zn in addition to Fe^10,44^. In this study, both Zn transporter-like (*LOC100037509*) and Zn transporter (*LOC101255999*) genes showed a substantial decrease in Ratan root expression patterns that could help to render the Zn root system inefficient for Zn regulation. On the contrary, the induction of ZIP transporter occurs in root or leaf, depending on the plant species/sensitivity^45^. These genes can be good candidates for further functional characterization of Zn-transporter in tomato through genome editing and yeast complementation assay.

The localization of Zn transporters was further validated by the computational prediction. The CELLO analysis predicts that all of these tomato Zn transporters are localized in the plasma membrane which is in agreement with wet-lab experiments reported previously in various plants^46,47^. Ion and solute fluxes underpin inorganic mineral nutrient uptake at the plasma membrane, thereby triggering changes in second messengers such as cytosolic-free Ca^2+^ concentrations in cell^48^. The interactome map further suggests that the Zn transporters in tomato are associated with some genes related to metal ion transporter and transcription factor. The Fe-regulated transporter 1 in tomato is in close partnership with metal transporter (*NRAMP1*, *NRAMP2*), Fe reductase gene (*FRO1*), *fer* transcription factor, and nicotianamine synthase (*CHLN*) genes. Previous studies also demonstrated that the *BHLH068* transcription factor via interaction with *FER* along with *IRT1, NRAMP1* and *FRO1* is involved in iron homeostasis in tomato^49^. Further, *CHLN* encodes nicotianamine forming a complex with Fe^2+^ for the distribution of Fe in plants^50^. The defect in *FER* mutant caused dramatic downregulation of Fe-uptake genes (*IRT1*, *FRO1*) in tomato; however, the molecular mechanism remains unclear^46^. However, *FER*-like transcription factor FIT, expressed in roots was associated with *FRO2* and *IRT1* genes in Arabidopsis^51^. In order to bind to the promoters of *IRT1* and *FRO2*, this *FIT* is not able to act alone but must form a heterodimer with other bHLH proteins, as shown in yeast^52,53^. It is, therefore, possible that Zn-deficiency is associated with the coordination of Fe homeostasis in tomato. Consistently, this study also suggests the involvement of *ZIP* metal ion transporter and *BHLH* transcription factor with Zn transporters in tomato. Overall, this interactome analysis might provide essential background for functional genomics studies in characterizing transporters and transcription factors underlying Zn-deficiency responses in tomatoes.

### Oxidative damage and antioxidant response

In this study, the O_2_^**.**−^ was higher in the roots than in the shoot but significantly decreased in the shoot due to Zn-deficiency, which may be linked to elevated SOD activity. Further, H_2_O_2_ is a key ROS indication substantially induced in tomato following Zn starvation. The increases in ion flux and gene expression are part of H_2_O_2_ in plants^43,54^. The degradation of elevated H_2_O_2_ into less reactive molecules is frequently correlated with CAT^55^. Hacisalihoglu et al.^56^ reported that Zn-efficiency is allied with the Zn-SOD enzyme in wheat. In this study, Ratan showed a consistent decline in antioxidant enzymes that are known to inhibit H_2_O_2_ in the shoot in response to Zn deficiency. Further, APX and GR also showed no induction in tomato subjected to Zn-starvation, suggesting that inefficiency to ROS mitigation is possibly related to the damage of chloroplasts and cellular proteins. Cells are also prone to ROS pools due to abiotic stresses, not just chloroplasts involved in energy production^57^. It implies that ROS scavenging is not present actively in this Zn-deficiency sensitive tomato, especially in the shoot, to combat Zn deficiency. Thus, it pinpoints that the increase of ROS and the decrease in chlorophyll synthesis in Ratan is correlated with the photosynthesis inefficiency in leaves. However, the ROS regulation in plant cells may vary on the species and cultivar of species under Zn deficiency.

## Conclusion

Zn deficiency showed a substantial reduction in plant biomass, photosynthetic efficiency, and cellular damage in Zn-deficiency sensitive cultivar (Ratan). The expression of iron-regulated transporter 1, zinc transporter-like (*LOC100037509*) and zinc transporter (*LOC101255999*) significantly decreased following Zn-deprivation resulted in a substantial decrease in Zn and Fe status in either root or shoot of tomato. Although the metal uptake in plants depends on many genes and the activity of the encoded protein, these two Zn-transporters may encourage further research regulating Zn-efficiency in tomato. Furthermore, the morpho-physiological retardation was consistent with the inefficiency of antioxidant defense to cope with the elevated oxidative injuries. This outcome will help to clarify our understanding of Zn deficiency more effectively to accelerate breeding or genome-editing program to improve Zn-deficiency tolerance in tomato for human nutrition.

## Supplementary Information

Supplementary Information

## Data Availability

All data of the manuscript are available.
