# Supplementary material for: Downregulation of Zn-transporters along with Fe and redox imbalance causes growth and photosynthetic disturbance in Zn-deficient tomato
Source: Sci Rep. 2021 Mar 16;11:6040. doi: 10.1038/s41598-021-85649-w (PMC7966403; doi:10.1038/s41598-021-85649-w)
Supplement: Supplementary file 1 — Supplementary Information [file 41598_2021_85649_MOESM1_ESM.docx]

**Downregulation of Zn-transporters along with Fe and redox imbalance causes growth and photosynthetic disturbance in Zn-deficient tomato**

Ahmad Humayan Kabir ^1,^*, Mst Salma Akther ^1^, Milan Skalicky ^2*^, Urmi Das ^1^, Gholamreza Gohari ^3^, Marian Brestic ^2,4^ and Md Monzur Hossain ^1^

^1^ Department of Botany, University of Rajshahi, Rajshahi 6205, Bangladesh; ahmad.kabir@ru.ac.bd (A.H.K.), salma94ru14@gmail.com (M.S.A.), dasurmi2911@gmail.com (U.D.), monzurhb@gmail.com (M.M.H.)

^2^ Department of Botany and Plant Physiology, Faculty of Agrobiology, Food and Natural Resources, Czech University of Life Sciences Prague, Kamycka 129, 165 00 Prague, Czech Republic; skalicky@af.czu.cz (M.S.)

^3^ Department of Horticultural Sciences, Faculty of Agriculture, University of Maragheh, Maragheh, Iran; gohari.gh@maragheh.ac.ir (G.G.)

^4^ Department of Plant Physiology, Slovak University of Agriculture, Nitra, Tr. A. Hlinku 2, 94901 Nitra, Slovakia; marian.brestic@uniag.sk (M.B.)

*Corresponding author:

ahmad.kabir@ru.ac.bd (A.H.K.), skalicky@af.czu.cz (M.S.)

**Table S1.** List of primer sequences used for quantitative Real-time PCR.

| **Genes** | **Accession number** | **Primer sequences** |
| --- | --- | --- |
| *Actin*  (Housekeeping gene) | NM_001323003.1 | F: ACATGCCATACTTTTCACCGC  R: CCAGCCTTGACCATTCCAGT |
| *Iron-regulated transporter 1* | AF136579.1 | F: TTGGTGCTGGTGGAATGTCA  R: AAGAAGGCCACACTTATTACTCA |
| *Zinc transporter-like (LOC100037509)* | NM_001247420.1 | F: GGATGATCCCAAATCGCCCT  R: ACCCCTTGAGTTGTGGCATC |
| *Zinc transporter (LOC101255999)* | NM_001322833.1 | F: GGGAGAGGCATGGAGAAACC  R: CAGCTGTACGGCCTTCACTA |
